# Refining core competencies of first-line nurse managers in the hospital context: A qualitative study

**DOI:** 10.1016/j.ijnss.2023.08.001

**Published:** 2023-08-10

**Authors:** Joko Gunawan, Yupin Aungsuroch, Mary L. Fisher, Colleen Marzilli, Nazliansyah, Ety Hastuti

**Affiliations:** a Faculty of Nursing, Chulalongkorn University, Bangkok, Thailand; b Indiana University School of Nursing, Indianapolis, IN, USA; c College of Nursing, University of Florida, Gainesville, FL, USA; d University of Maine, School of Nursing, Orono, ME, USA; e Department of Nursing, Politeknik Kesehatan Kementrian Kesehatan Pangkal Pinang, Bangka Belitung, Indonesia; f dr. H. Marsidi Judono General Hospital, Belitung, Indonesia

**Keywords:** Clinical competence, Core competencies, Head nurses, Indonesia, Social skills

## Abstract

**Objectives:**

As the world moves towards a new normal, challenges continue to emerge while simultaneously inspiring us with new solutions. Strengthening the skills of first-line nurse managers (FLNMs) to fulfill a wide range of complex roles and responsibilities effectively necessitates refining core competency guidelines or standards. This study aimed to explore the perceived core competencies of Indonesian FLNMs within the context of the post-pandemic era.

**Methods:**

The study employed a qualitative descriptive design. Face-to-face interviews were conducted in a public hospital in Indonesia from January 2022 through August 2022. Seven head nurses with direct experience managing a unit during the COVID-19 pandemic were selected. The interviews were audio-recorded, transcribed verbatim, and validated by re-listening. Data were analyzed using thematic analysis.

**Results:**

Four main themes of the core competencies developed, including 1) managerial core competencies, 2) clinical core competencies, 3) technological core competencies, and 4) socio-emotional skills/personal traits consisting of the following: be brave, fast, patient, optimistic, consistent, and responsible.

**Conclusions:**

The findings demonstrate that the managerial and clinical core competencies of the FLNMs must be aligned, while technological core competencies are the mediating component of both. Personal traits are essential for FLNMs as they undergird the other three core competencies and the success of the FLNMs.

## What is known?

• The COVID-19 crisis has emphasized the importance of first-line nurse managers acquiring additional skills, such as managing hybrid workflows, efficiently allocating human resources during emergencies, and implementing innovative leadership strategies.
• While several studies have explored the core competencies of nurse managers, there has been a shortage of specific focus on first-line nurse managers. Furthermore, these studies have predominantly been conducted before the pandemic and within country-specific contexts.
• Given the ongoing transition towards a new normal, refining the core competencies of first-line nurse managers in the hospital context is crucial to align with the evolving demands and challenges of the post-pandemic era.

## What is new?

• The findings of this study offer valuable insights and serve as a foundation for hospital managers and nurse managers in developing their leadership plans, designing effective training and development programs, and creating assessment tools to evaluate the core competencies of first-line nurse managers.
• The identified core competencies, encompassing managerial, clinical, technological, and socio-emotional skills/personal traits, contribute to the existing body of nursing knowledge concerning the core competencies specific to first-line nurse managers. This research holds particular relevance in the context of post-pandemic situations, where the roles of first-line nurse managers have become more significant and multifaceted.

## 1 Introduction

Core competencies, defined as a set of skills, attributes, and behaviors necessary for employees in a specific role, significantly contribute to the effective performance of job responsibilities [1]. For first-line nurse managers (FLNMs), core competencies encompass a combination of skills, knowledge, attitudes, and behaviors that enable them to navigate the complexities of the healthcare system, adapt to changing circumstances, and ensure the delivery of high-quality care for the success of healthcare organization [2,3].

The COVID-19 pandemic has significantly transformed various aspects of society over the past three years and profoundly impacted FLNMs. In addition to the fundamental changes in working, socializing, and mobility, FLNMs have faced unique challenges arising from the pandemic and the emergence of new events and diseases [4]. This double burden has necessitated developing and enhancing core competencies among FLNMs to effectively manage these complex and evolving circumstances while adapting to different environments, maintaining care quality, and fulfilling hospitals’ missions and visions [5]. As the world transitions toward an endemic phase of COVID-19, FLNMs should prepare themselves by improving their core competencies and equipping themselves with the necessary support available to navigate the new normal conditions. The term “new normal” refers to the need to adapt to new ways of living and working since the world will not return to how it was before the pandemic. Therefore, FLNMs must be ready to face these new challenges and adapt to changing conditions [4].

Acknowledging the shifting roles and responsibilities of FLNMs, it becomes evident that the core competencies required to excel in their positions have also evolved. While existing core competencies may have been applicable before the pandemic, the COVID-19 crisis has highlighted the need for FLNMs to acquire additional skills, such as managing hybrid workflows, quickly allocating human resources in emergencies, demonstrating caring for their staff in offline and online settings, and implementing innovative leadership strategies amidst uncertainty, resource constraints, and increased patient loads [6]. Furthermore, FLNMs have often found themselves directly involved in patient care [7].

FLNMs worldwide have implemented various strategies to address the challenges brought about by the pandemic effectively. For example, in Jordan, approaches included establishing support teams, field hospitals, and new units and modifying emergency plans and policies [8]. In addition, efforts were made to enhance medical supplies, personal protective equipment (PPE), and safety standards, alongside providing virtual training for staff. These experiences enhanced leadership competencies and emphasized self-awareness and personal growth [8]. Similar initiatives were observed in Egypt [9] and Spain [10], equipping FLNMs with disaster management competencies, ethical decision-making abilities, and competencies to navigate uncertainty and prioritize staff well-being. In addition, Iranian nurse managers required flexible and situational management to recruit, retain and compensate nurses [11] and emphasized the religious spirit as they served as role models for their staff during the pandemic [5].

Furthermore, regarding the current literature on core competencies, a recent study [3] identified eight essential competencies for nurse managers, including “decision-making, relationship management, communication skills, listening, leadership, conflict management, ethical principles, and collaboration and team management skills.” However, the study did not differentiate between the core competencies of different levels of nurse managers and focused on all nurse managers in the Spanish health system. Additionally, a scoping review [12] synthesized the most-cited competencies of nurse managers into 22 most-cited competencies, but these competencies were not specific to FLNMs. Moreover, a study in Ghana [13] examined essential management competencies based on Katzʼs model, which assessed technical, human, and conceptual skills without exploring other core competencies in the current context.

Therefore, it remains unclear whether the core competencies identified in previous studies adequately capture the core competencies required for FLNMs in Indonesia and best fit todayʼs new normal. This raises important questions: Are the core competencies dimensions of FLNMs essentially different among countries or specific contexts? Are these skills necessarily required, or can we retain the same core competencies as before the pandemic? These cannot be answered without further evidence and validation.

To our knowledge, few published researches specifically examined FLNMsʼ core competencies as perceived by Indonesian nurse managers during and post-pandemic. While two local studies explored related topics, such as staff scheduling [14] and hospital preparedness during the early stages of the pandemic [15], they did not directly address FLNMs’ core competencies. Therefore, further investigation into this area is warranted.

To bridge the gap, our study aimed to explore the core competencies of Indonesian FLNMs within the context of the new normal. The research question for this study was “What are the perceived core competencies of Indonesian FLNMs in the post-pandemic era?” Understanding the core competencies in this context will inform the development of tailored standards and guidelines that empower FLNMs to effectively navigate future challenges, ensuring high-quality care and resilience in the face of uncertainties.

It is noteworthy that, although in international contexts, FLNMs are referred to by various titles such as ward managers, head nurses, ward sisters, unit managers, and charge nurses [2]; however, in Indonesia, a first-line nurse manager refers to a clinical nurse holding positions such as head nurse, ward manager, ward head nurse, or unit manager. Charge nurses and ward sisters are not considered equivalent to FLNMs. The first-line nurse manager is known as “Kepala Ruangan” in Indonesian [2].

Moreover, it is common for the terms “first-line nurse managers” and “front-line nurse managers” to be used interchangeably, describing nurses in managerial positions responsible for overseeing daily operations within a specific unit. Generally, both terms denote the exact role of nursing management at the frontline. The variation in terminology can depend on the context, with “first-line nurse managers” emphasizing the hierarchical position and “front-line nurse managers” highlighting their direct involvement in patient care. In Indonesia, nurse managers are categorized into different levels within the management hierarchy. First-line nurse managers are classified as low-level managers, while middle managers include area managers and heads of sub-nursing departments. Top-level managers comprise the heads of nursing departments and directors or vice directors of nursing [2]. Therefore, to align with the specific context of our study and maintain consistency, we exclusively utilized the term “first-line nurse managers” (FLNMs) throughout the article.

## 2 Methods

### 2.1 Study design

This study used a qualitative descriptive design as outlined by Lambert and Lambert [16]. The design was a suitable choice for our research as it facilitated a direct and straight description of the phenomenon, especially the core competencies of Indonesian FLNMs. This design collected data from FLNMs and analyzed thematically, allowing for a comprehensive exploration of the core competencies. The focus was on providing a descriptive summary of the findings without requiring extensive theoretical competency development [16]. This approach ensured that the study remained grounded in the participants’ experiences and perspectives, providing a clear and concise account of the FLNMs’ core competencies. There was no manipulation or commitment to any theoretical view in this design, as there was no philosophical or epistemological strand. The findings were purely derived from the data codes, reflecting the naturalistic inquiry [16]. A qualitative descriptive design was selected over a grounded theory design as the constant comparative analysis was employed, but no theory was developed. In addition, the qualitative descriptive design was chosen over a phenomenological approach because it included less interpretive illustrative components [16].

### 2.2 Ethical consideration

The study approval was obtained from the Research Ethics Committee, Department of Health of Indonesia, Indonesia (Ref code: 800/0375/RSUD dr. H.M.JD, approved on January 27, 2022). Study permission was also given by the hospital. Participants had to sign an informed consent with the stated study objectives to participate in this study. It is noted that this study was voluntary, and participants could withdraw from participation after data collection was concluded. Confidentiality of the data was highly maintained and stored in a secured computer. The researchers did not use real names in reporting the study. Instead, numbers were used (P1–P7).

### 2.3 Participants

Seven FLNMs were selected using purposive sampling. The advantage of purposive sampling is to enable researchers to obtain rich information from those who understand a phenomenon [17]. The participants’ inclusion criterion was FLNMs who had direct experience managing a unit during the COVID-19 pandemic, specifically since the early stage of the crisis in Indonesia (from March 2020), to provide the actual phenomenon and details in the field, based on their perspectives.

### 2.4 Data collection

Data were collected from January 28, 2022 to August 10, 2022, in a public hospital in Belitung, Indonesia, after obtaining the study permit. The top nursing manager, as directed by the hospital director, assisted the researchers by providing the names and phone numbers of the FLNMs who had direct experience managing a unit during the COVID-19 pandemic. Subsequently, the researchers contacted the FLNMs through short message service (SMS) and phone calls. Additionally, the middle-line nurse manager contacted the FLNMs to encourage their participation in the study. All FLNMs, according to inclusion criteria, were invited to participate, and those who were available and accepted the invitation were scheduled for an interview at a mutually agreed upon time.

Semi-structured face-to-face interviews were the primary method for data collection. The interviews were audio-recorded, transcribed verbatim, and validated by re-listening. The interviews for this study were conducted by three researchers, namely NN, JG, and EH. Based on their convenience, the interviews took place either in a meeting room at the hospital or at the participant’s home. However, it should be noted that EH, who held a middle-line nurse manager position, participated in the interview only once. This decision was made to minimize potential response bias, as EH had a professional and hierarchical relationship with participants from the same institution. It is worth noting that NN and JG had no prior connection or significant relationship with the participants, as they were from different organizations, ensuring impartiality during the interviews. The interviews were conducted in Bahasa, or the Belitung language, to facilitate effective communication with the participants.

The interview questions can be seen in Table 1. There was no pilot testing of the interview guideline, as the questions were only for initial probing questions. In addition, the researchers also used chats, like WhatsApp, as an additional data collection method. However, this method was limited to clarifying the participants’ responses if the recording was unclear during verbatim transcription. One participant sent her voice to answer the interviewerʼs questions through WhatsApp. The researchersʼ mobile phones automatically recorded the chats while the voices were transcribed verbatim and re-read. It should be noted that combining data collection from various sources in a single study yields reliable and trustworthy findings [18].

**Table 1:** Semi-structured interview questions.

Semi-structured interview questions	
General questions	• What kind of core competencies do you think we need as FLNMs in the COVID-19 pandemic, and what do we learn for the new normal? Can you please explain? • Why are the core competencies that you mentioned necessary? • What core competencies are priorities for you? Why? • Can you sort those core competencies? Why?
Specific questions to elicit responses	• Is leadership included in a list of core competencies of FLNMs in the COVID-19 pandemic and new normal? What kind of leadership do we need, and what is expected? Why? • Is human resource management included in the core competencies of FLNMs? Why? What kind of human resource management? • Is technological skill included? Why? What kind of technological skills do we need? How to apply technology to your work? What type of technology do you expect? • Is clinical skill included in a list of core competencies of FLNMs? Why? Don’t you think the FLNMs only focus on management and administration? • Is political skill needed, and should it be included in a list of core competencies? Why? How? • What are other core competencies needed according to your experience during COVID-19 and beyond? This will be important because we don’t know what kind of disasters or emergencies we may face in the future, so we can better prepare. Please explain!

The process was concluded when data were saturated occurred. It is noted that a content analysis of the interview data of each participant was done before the following interview. Therefore, the researchers/interviewers noticed if another participant provided the same responses.

The interview averaged about 50 min for each participant. In addition, there was one repeated interview (P3), and chats via WhatsApp were conducted several times to clarify the participants’ answers.

### 2.5 Data analysis

A thematic analysis following Vaismoradi, Turunen, and Bondas [19] was employed. The process was comprised of 1) transcribing each interview, 2) re-reading the transcripts several times and highlighting the codes that have meaning units, and 3) developing subthemes and themes by organizing, comparing, and contrasting the meaning units [19]. This fundamental analysis relied on the explicit description with a lower interpretation level rather than the implicit meaning with a more profound interpretation [19,20]. The findings were reported with no further theoretical analysis. Therefore, this thematic analysis was suitable for the qualitative descriptive study design.

Manual data analysis was done for this study. First, JG, NN, and EH performed initial transcription (verbatim), coding (144 codes), and developing categories, subthemes, and themes generated from the codes in the Indonesian language from August 10 to 30, 2022. Next, all themes and participants’ quotes were translated into English, and the first drafts of the study report and manuscript were developed from August 30 to September 20, 2022. A draft manuscript containing initial themes and all quotes was then shared with the research team on September 20, 2022, and their feedback was used to refine the results.

The framework of Abfalter, Mueller-Seeger, and Raich [21] was utilized for the translation decisions, comprising seven elements. The first question consisted of why. The why was to develop scientific value among researchers and for dissemination to advance FLNM core competencies. The second question consisted of when. The when was from the initial draft development through the end of the study. The translation in the initial findings allowed all researchers to look at the data and identify if additional interviews were needed. The third question is what. The what consisted of interview data and all reports. The fourth question is who. The who consisted of the interview data and drafts that were translated by JG, NN, and EH. The fifth question is how. The how is JG did the translation from Bahasa to English, and NN and EH compared both versions for clarity. The sixth question is where. The where is within the English-speaking research team environment. The seventh question is by what means. The by what means is that language proficiency was ensured and no IT applications were used.

### 2.6 Rigor and trustworthiness

Multiple strategies were implemented to ensure rigor and trustworthiness in this study. Firstly, a peer review process involving nursing experts from the research team and an external researcher was conducted within two weeks to assess authenticity, objectivity, and credibility while ensuring the absence of bias or preconceived notions during the analysis and theme development stages. Secondly, member checking was performed on August 30, 2022, allowing participants to review and confirm the thematic findings. Thirdly, the emerging codes, categories, and themes were checked and validated by all researchers through consensus. Fourthly, the research design, implementation, and data collection process were thoroughly described to ensure dependability. Finally, the researchers acknowledged their own positionality and potential biases, with one researcher (EH) minimizing her involvement in most interview sessions and participating in only one interview to mitigate bias.

## 3 Results

The participants in this study consisted of five females and two males aged 34–46 years. They have an average of four years’ experience as an FLNM with a maximum of six years’ experience. Their experience with COVID-19 as an FLNM included working in isolation units, emergency units, ICUs, internal medicine, airborne and pulmonology, and perinatology care areas. In addition, the participants’ education levels comprised bachelor’s and diplomas in nursing, with clinical experience between six to 14 years. The detailed information can be seen in Table 2.

**Table 2:** Demographic characteristics of participants.

Participant No.	Age	Sex	Unit/Ward	Education	Experience to be FLNM (years)	Clinical nurse experience before being FLNM (years)
P1	38	Female	Air Borne and Pulmonology	Diploma in Nursing	6	10
P2	38	Female	Internal Medicine	Diploma in Nursing	6	10
P3	43	Female	Isolation	Diploma in Nursing	4	8
P4	34	Male	Emergency	Bachelor in Nursing	4	6
P5	46	Female	Perinatology	Bachelor in Nursing	6	14
P6	41	Male	Surgical Medical	Diploma in Nursing	6	11
P7	35	Female	Isolation	Diploma in Nursing	5	8

Four main themes of the core competencies developed: 1) managerial core competencies, 2) clinical core competencies, 3) technological core competencies, and 4) personality traits. Notably, researchers did not anticipate the emerging themes before the study. The themes are presented with exemplary quotes from the participants, with Participant No. (P1–P7) and Codes (C).

### 3.1 Theme 1: managerial core competencies

Managerial core competencies refer to the primary set of skills, abilities, attributes, and behaviors required for FLNMs to implement particular managerial responsibilities. Seven subthemes were developed in this theme: 1) human resource management (with four categories: staffing, rewards, training, and performance evaluation), 2) self-management, 3) supply and equipment management, 4) patient care management, 5) knowledge management, 6) leadership, and 7) unit budgeting. The detail of each subtheme and exemplar quotes can be seen in Table 3.

**Table 3:** Subthemes of managerial core competencies and exemplar quotes.

Subthemes/Dimensions/Items	Exemplar quote
**(1) Human Resource Management**	
***Staffing***	
Calculating the number of nurses based on the need	• *“We should be able to calculate the need for nurses with the number of patients. Donʼt let the nurse get tired. For example, eight patients, eight nurses, and one doctor.”* (P2, C12) • “*For human resource (HR) management, yes, it is adjusted to the number of patients based on the need …*” (P5, C93)
Taking part in selecting nurses for the unit based on skills, qualifications, attitude, knowledge, merit, and personality	• *“For the selection of nurses, actually, the hospital has employee mapping, which one is appropriate to be included in the unit, particularly in our unit (Emergency Room, ER). For example, three years of experience as a clinical nurse, emergency training certification, and required skills. If they do not meet the requirement, we will reject them.”* (P4, C49) • *“We must be able to select competent nursing staff, in this case, the responsive ones, both to patients and nursing documentation. This is an additional requirement to the physical criteria.”* (P2, C20)
Managing nurse workforce diversity and building teamwork between senior and junior nurses	• *“Junior and senior nurses work in a team, and sometimes there are obstacles between the two. This is actually not only a matter of being strong or not but also a mindset. Sorry, there used to be a lot of senior nurses who were absent often. So, I try to facilitate them. In the worst case, I need to rotate them.”* (P4, C50) • *“In our standard, a nurse’’s age should be between 23 and 35. If you are above 35, sometimes many people complain of being sick. But some still survive because their physical is still supportive and capable. So, sometimes it has to be adjusted to the condition. It is also useless if you select a younger nurse who cannot do anything or lacks skills. Junior, senior nurses must work in a team indeed.”* (P4, C55)
Scheduling nurses’ shifts with flexibility, transparent and objectively	• *“The nursing workload in the ER is the same for every shift. So, if a nurse gets sick, there is no need to force them. It can be rescheduled with others. If it is not possible, the nurse can ask for rotation … for the health of the nurses themselves and the continuity of the service.”* (P4, C56) • *“… for the shift schedule, it’’s the same, flexible to help each other.”* (P5, C93)
***Rewards***	
Setting up a reward system for the unit if it is not available for staff nurses	• *“Rewards at the hospital are only for the head nurses. So, I make my own unit system for the staff. Mostly the rewards I received from the hospital were shared with my subordinates in accordance with the system that was created.*” (P5, C87)
Rewarding staff nurses based on performance	• *“At the hospital, there is also a reward for change agents. So, the head nurses choose team leaders of shift chairs to be role models, and they are proposed to the hospital. The selection is based on their work, and indeed, they need appreciation.”* (P5, C89)
Providing monetary rewards	• *“We ever gave a reward, like 200,000 IDR, to motivate them (nurses) every month …”* (P4, C59)
Providing non-monetary rewards (recognition, reinforcement, etc.)	• *“In the unit, we have a reward “star of the month” seen from discipline and attitude.”* (P4, C58) • *“There are also gifts for the subordinates, like when the birthday celebration.”* (P5, C88)
***Training***	
Assessing training needs for staff nurses	• *“We assess the need for training of the staff. We need to keep updated.”* (P4, C64)
Setting up training opportunities or programs (workshops, seminars, conferences) inside and outside the hospital	• *“During this new normal, the nurses need a leader that can protect and facilitate nurses, including providing training as well as case reflection meeting). We also need to set benchmarking apart from training to advance our service by learning from others.”* (P4, C67)
Being able to be a trainer or a mentor for staff nurses	• *“If there are new nurses, as head nurses, we need to be able to direct them, train and mentor them properly.”* (P7, C127) • *“… a new problem arises; when nurses are rotated, their competence is lacking. For instance, stitching the wound, inserting NGT, and dealing with case accidents. Indeed, we need to train them again. We must help each other.”* (P4, C47)
Providing a preceptor training program for staff nurses in order to guide new nurses and nursing students	• *“… we must also be able to guide nursing students who practice in the unit. Many students cannot do basic techniques, such as inserting an infusion. In fact, they almost graduate. On the other hand, the head nurses should appoint competent nurses to teach students. Although sometimes, head nurses need to train nurses, like preceptor training, as well as how to teach students.”* (P4, C74)
Providing comprehensive training for staff nurses (clinical, management, and emotional training)	• *“Spiritual, emotional training is necessary, in addition to clinical and management training.”* (P4, C70)
***Performance evaluation***	
Evaluating staff nurses’ performance regularly	• *“Head nurses should be able to evaluate staff skills. The standard operational procedure already exists …”* (P6, C108) • *“Ongoing professional evaluation I do per semester to the staff. The evaluation form is available in each unit.”* (P5, C90) • *“We, head nurses, observe the staff performance directly”* (P4, C59)
Assessing staff nurses’ performance objectively according to hospital standard	• *“Performance, ethics, and competence are to be evaluated. All aspects should be measured and presented using a percentage …”* (P5, C91a)
Communicating the evaluation results to staff nurses; Suppose having poor performance, individual communication will be done (assessing the weakness, attending training, or being rotated to another unit); Suppose having a good performance, rewards will be given accordingly	• *“… and we continue to communicate with nurses, either individually or through pre-post conferences.”* (P5, C91b) • *“… if nurses are often absent, we really have to ask them directly, what they actually want, to ask for the best solution.”* (P4, C50)
Working closely with the hospital nursing committee to communicate the results of staff performance evaluation for career planning (a part of the credentialing process)	• *“Credentials are only for clinical nurses’ level, while non for another level, such as manager level. However, we still need to coordinate closely with the credential team to evaluate staff and develop the credentialing process.”* (P5, C76)
**(2) Self-Management**	
Knowing self/self-awareness/self-reflection regarding personal strengths and weakness	• *“The ability for self-reflection is also a necessity … so we don’’t blame each other if there is a problem.”* (P4, C69) • *“If a leader is excellent, the staff also follow. We need to know our own strengths and weakness.”* (P7, C123)
Managing self to attend training/workshop/seminar/conference (informal education) based on the need	• *“Self-development is needed. Like earlier, I attended speaking training, communication, and self-training.”* (P4, C63) • *“To improve self-quality, yes, I join the training outside.”* (P5, C83)
Developing self through formal continuing education	• *“Many nurse managers here still hold diploma 3 nursing … continuing formal education is a necessity.”* (P5, C78)
Asking for feedback from top management regarding performance	• *“The evaluation system in this. So, all managerial and clinical activities are evaluated.”* (P5, C98) • *“Assessors for nurse managers are not yet available.…management functions, like planning itself, have never been evaluated by top managers (heads of nursing section and department).”* (P5, C79, C105)
Asking for opinions from staff nurses regarding leadership and management	• *“Sometimes I ask my staff to give me input or feedback on what kind of head nurses they need.”* (P5, C85)
Participated in professional nurse associations and professional development programs	• *“It would be much better if head nurses could join a professional organization like PPNI (Indonesian National Nurses Association) or HPMI (Association of Indonesian Nurse Managers) to improve our knowledge.”* (P5, C94b)
Being engaged in a lifestyle that fosters physical, mental, and spiritual well-being	• *“During the pandemic, I had a lack of sleep. But in this new normal, we change, live healthily, with a good lifestyle, always wear a face mask, and bring hand sanitizer everywhere.”* (P7, C131) • *“From COVID-19, we learned about healthy life and always washing our hands. Always change clothes every time entering the unit. Wear a mask, and manage eating time and sleep quality. We should be healthier.”* (P5, C94c)
**(3) Patient Care Management**	
Managing patient care coordination (multidisciplinary) in the unit	• *“We need to be able to coordinate with medical doctors and others about medication management.”* (P3, C31) • *“During the pandemic, head nurses should be able to coordinate with the Department of Health and Regional Government. The communication with them using WhatsApp, either for reporting new COVID-19 cases, patients in care, or discharged patients.”* (P3, C32) • *“Head nurses also need to collaborate with the nutrition department. So, the staff from that department visit this unit every morning to see every patient*’*s medical record.”* (P3, C36) • *“Collaboration with pharmacists usually via WhatsApp as well.”* (P3, C37)
Facilitating collaboration between nursing staff and other health professionals, especially physicians, in providing patient-centered care	• *“… we also need to facilitate our staff’’s collaboration with other healthcare professionals …”* (P3, C38)
Describing the clear process of intra and interprofessional collaboration and the roles and practices of effective teamwork, particularly to avoid overlapping job descriptions	• *“… we need to understand our own competency in order to prevent overlapping with other professions. For example, regarding the mixing of drugs, the pharmacist should be the one to do it. Similar to taking lab samples, which the lab staff themselves should do. So, delegation, coordination, and communication must be clear.”* (P5, C97)
Involving family, friends, and alternatives in patient care	• *“As head nurses, we can do health education not only for patients but also for their families.”* (P3, C33)
Participating in developing a data-centric, integrated, and comprehensive care plan	• *“This hospital needs a full integration with the available technological system because many things are still done separately. All nurse managers should participate in that integration thing.”* (P6, C111)
Holding self-accountable to comply with ethical standards of practice and policy	• *“Besides, in the service, we continue maintaining services according to the hospital and nursing code of ethics, including the rights of patients themselves.”* (P7, C132)
Ensuring all nurses follow ethical principles and protect client’s autonomy, dignity, and rights	• *“We also need to ensure our staff follows the hospital and nursing code of ethics because patients need to be treated well.”* (P7, C133)
Assessing the quality of patient outcomes regularly	• *“… from audit, service quality is also evaluated. Yes, our service quality should be assessed periodically …”* (P5, C103)
**(4) Knowledge** M**anagement**	
Being able to find and clarify new knowledge, evidence, or new information for nursing practice	• *“Knowledge is important. We have to understand the problem, like COVID-19.”* (P7, C113) • *“Many say COVID-19 is dangerous and deadly. Many also stay away from nurses because of COVID-19. Head nurses must be patient, optimistic, and ready to educate staff and the public about this issue. We must have good knowledge.”* (P7, C117)
Sharing the knowledge offline and online	• *“We used Zoom meetings during COVID-19, meeting every two months. There were case discussions and sharing of knowledge from medical doctors or others, for example, about knowledge that is still trending. Sometimes I also become a speaker.”* (P5, C82)
Applying the knowledge to practice	• *“… from the knowledge we get, we try to apply it in the unit.”* (P5, C82a)
Evaluating the outcome	• *“… and then we assess the outputs from the knowledge we get.”* (P5, C82b)
**(5) Leadership**	
Being a role model	• *“Head nurses must look capable of being role models for staff, not only able to take care of administration.”* (P1, C2) • *“… become a role model for subordinates, such as arriving on time, and being ready at any time when the subordinates need us, and being prepared to sacrifice.”* (P2, C17)
Managing working problems and conflicts among staff	• *“… able to solve problems, both in management and clinic …”* (P2, C18) • *“Conflict management is necessary, and we must know the causes. This unit has 45 staff, including nurses, midwives, and doctors. If there is a problem, we need to solve it. Call those involved, ask, and collect more data before making decisions. Mostly we solve the issues internally.”* (P4, C48) • *“Leadership technique is needed, especially for managing conflict between subordinates.”* (P6, C109)
Involving staff in shared decision making	• *“Cohesiveness between the head nurse and staff is necessary. Sometimes I share with my staff before making a decision.”* (P5, C106)
Having an adaptable leadership style according to staff autonomy	• *“Leadership is essential to manage the staff and unit. The leadership style used is based on the staff, but mostly I use democratic leadership.”* (P2, C16) • *“The type of leadership I apply according to my subordinates, whether they are independent or not. If they are independent, we can just simply direct them.”* (P5, C85)
Being able to direct and empower staff	• *“… directing staff, because they actually are competent already.”* (P1, C4) • *“If there are new nurses, we must guide them, teach and direct them. We need to be firm, especially during the pandemic.”* (P7, C127) • *“We must be able to be a consultant for subordinates. Often, my staff asks me about skills and medication.”* (P3, C29)
Having effective interpersonal communication	• *“The communication strategy to use is effective communication. There is effective feedback with staff …”* (P4, C65) • *“Head nurses should have advanced communication skills, both with staff and patients.”* (P6, C107) • *“… it means the role of good communication. How to speak well with patients and the community. For example, many patients are stressed after contracting COVID. The communication role is important here.”* (P7, C118)
Having political and negotiation skills, particularly in selecting nurses, approving the budget, implementing a program, and adjusting patient care.	• *“Political and negotiation skills. For example, the capability to include and transfer nurses to achieve goals with existing human resources.”* (P1, C8) • *“Negotiation skill is highly needed. If the supply and equipment are lacking, we need to do lobbying to our superiors.”* (P5, C92) • *“Lobbying skill is certainly necessary, especially to select nurses for the unit.”* (P7, C130)
**(6) Supply and Equipment Management**	
Identifying and planning supplies and equipment needed in the unit	• *“As head nurses, we need to be able to calculate supply and equipment or facilities in the unit.”* (P2, C13) • *“The equipment in this hospital is already advanced, actually. USG (ultrasonography), ventilator, transport mobile, and ambulance are complete. But we need an emergency ambulance … because we now have only an ambulance for transportation.”* (P4, C62) • *“Not only human resources, head nurses also need to think about and plan facilities in the unit …”* (P5, C80)
Maintaining the records and reports of the supply and equipment, having inventory control, and keeping buffer stock for emergency	• *“For equipment, we just do the inventory and keep the record and quality.”* (P4, C62b) • *“In the unit, we need to maintain the equipment together. We check which one is still good or needs to change. Supply stock is also needed to check, especially during a pandemic.”* (P4, C74b)
Distributing material adequately for morning, evening, and night shifts	• *“We also need to be able to divide supplies and equipment in each shift in the unit.”* (P4, C74c)
Communicating “out of stock” supplies or non-functioning materials to staff and superiors	• “If the supply and equipment stock in the unit runs out, we will notify the installation department for follow-up.” (P4, C74d)
**(7) Unit Budgeting**	
Planning and budgeting for equipment and supply (short-term and long-term) in the unit	• *“We still make unit budgeting for supply and equipment in the unit and evaluate which ones are lacking and need more additional stuff, either for short or long terms.”* (P5, C99a)
Seeking to be involved in annual hospital budgeting	• *“Hospital planning should be done together, including budgeting. Sometimes what has been planned and the things we receive are different.”* (P5, C99)
Communicating expectations and outcomes to staff	• *“… What is given by the hospital, I communicate with my staff. Don’t let there be any misunderstanding.”* (P5, C99b)

### 3.2 Theme 2: clinical core competencies

Clinical core competencies are the sets of skills, abilities, attributes, and behaviors for FLNMs to provide safe care and achieve patient outcomes under particular circumstances within a clinical context. Learning from the pandemic, most participants agreed that they needed to have additional clinical skills. These skills must be advanced to accurately access and critically think about the best options for patient care. Six core competencies were identified: 1) emergency and critical care, 2) hospital disaster/crisis management, 3) case management, 4) infection control and prevention, 5) basic clinical skills, and 6) medication management. The exemplars of participants’ quotes can be seen in Table 4.

**Table 4:** Dimensions of clinical core competencies and exemplar quotes.

Dimensions	Exemplar quote
Emergency and Critical Care	• *“Not only nurses but head nurses also must have emergency skills, like intubation. Head nurses sometimes need to be in the shit when nurses are not enough, especially during the pandemic. So, critical care and emergency skills are required.”* (P3, C28) • *“Head nurses should have clinical competencies such as respiratory system skills, emergencies, transfusion skills (blood/plasma), and wound care.”* (P1, C1)
Hospital Disaster/Crisis Management	• *“… even if there is another pandemic or not in the future, it is clear that head nurses should have skills like disaster/crisis management, case management, as well as infection control and prevention.”* (P4, C73) • *“Disaster management! Yes, we need it. We can’t have too much workload again like the first time of COVID-19. Too stressful. This skill is not only for head nurses but all staff in the hospital. This is a system, not a personal matter.”* (P7, C137)
Case Management According to Diseases and Units	• *“… yes, case management to assess, plan, implement, and monitor patients from admission until they go home. And during COVID-19, we should ensure not to spread the contagion and treat patients best whatever it takes.”* (P7, C136)
Infection Control and Prevention	• *“… must have the knowledge, especially about the COVID case itself.”* (P2, C11) • *“Also, we need to understand the PPE concept, how to use it properly … how we can help others if we are first exposed to COVID.”* (P2, C15) • *“… even if there is another pandemic or not in the future, it is clear that head nurses should have skills like disaster management, case management, as well as infection control and prevention.”* (P4, C73)
Basic Clinical Patient Care	• *“Basic skills also are needed, like oxygenation, elimination, eating and drinking, and other basic clinical skills.”* (P2, C10)
Medication management	• *“Head nurses must also understand medication management and safety, such as drips, doses, and patient drug calculations.”* (P3, C30)

### 3.3 Theme 3: technological core competencies

Technological core competencies refer to the skill sets of FLNMs in understanding, using, and developing technology. Understandably, the daily management and clinical tasks the FLNMs perform rely on different tools and processes. In other words, technology is a mediating and essential variable in nursing management and practice; thus, enhancing technological skills is a necessity. The participants’ quotes can be viewed in Table 5.

**Table 5:** Dimensions of technological core competencies and exemplar quotes.

Dimensions/Items	Exemplar quote
Understanding and participating in developing and using technology in *clinical nursing practice and patient care*, especially for technological equipment and the use of multiple media for the patient care system	• *“… ability to use new medical devices …”* (P1, C7) • *“Now all systems are mostly online. For example, if the patient wants to go to the hospital for control, how we help them if we don’t understand the online system?”* (P2, C22) • *“… also includes the ability to report documentation online.”* (P2, C23) • *“For technology, it’s actually more about using technology itself. For example, from cellphones, we can play songs or music in the unit so the patient will be calm, like music therapy. So, it is enough with simple technology, but its utilization should be maximized.”* (P7, C128)
Being involved in creating and utilizing technology in *management practice*, particularly in interpersonal communication	• *“… able to use information technology to search for correct and valid information.”* (P1, C5) • *“We have a WhatsApp group for communication among nurses in the unit. So, if there is a piece of new information, we share it.”* (P2, C26) • *“… collaboration with pharmacists, usually via WhatsApp.”* (P3, C37) • *“We used Zoom meetings during COVID every two months. There were case discussions and sharing of knowledge from medical doctors or others …*” (P5, C82)
Participating in *data integration* through interoperability of electronic health records and supporting paperless	• *“Medical records are still manual. There are still many shortcomings. Sometimes the recording paper slips, and sometimes paper runs out. We need to support the system to be fully online and integrated.”* (P5, C101) • *“The technological ability needed here is more likely related to the online reporting system to the Department of Health.”* (P3, C35) • *“Reporting mortality cases using an application called SIMATNEO – maternal neonates, which is integrated into the Ministry of Health.”* (P5, C103)
Enabling internet access to staff nurses for data input and search information	• *“Lack of internet access, although there is a computer in the unit. We know we need to search for information and enter data online into the application. So yeah, we are on our own.”* (P5, C102)

### 3.4 Theme 4: personal traits

Learning from the pandemic, personal traits are needed to deal with complex environments and emergencies. Personality traits refer to FLNMs’ characteristic patterns of thoughts, feelings, and behaviors. These include being brave, fast, patient, optimistic, consistent, and responsible. These personal traits must be included in the managerial or leadership competencies, but we have differentiated between managerial core competencies and personality traits for clarity. The participants’ quotes are displayed in Table 6.

**Table 6:** Dimensions of personality traits and exemplar quotes.

Dimensions	Exemplar quote
Be Brave	• *“Head nurses and nurses themselves must have courage. Because, without it, it is hard to deal with COVID. Many are competent and skillful, but it’s complicated if they don’t dare.”* (P7, C119)
Be Fast	• *“In case of an emergency, without a leader who is quick to respond, the services will be neglected.”* (P7, C122) • *“What distinguishes the conditions before and during the pandemic is that the head nurses must be more active, fast, and agile to manage personnel and evacuate patients.”* (P4, C71)
Be Patient	• *“Many also stay away from nurses because of COVID-19.”* (P7, C115) • *“Head nurses must be patient, optimistic, and ready to educate staff and the public about this issue.”* (P7, C116)
Be Optimistic	• *“I have to stay optimistic about myself. Because the staff follows me. If I am not, the staff will be the same.”* (P5, C106b) • *“Head nurses must be patient, optimistic, and ready to educate staff and the public about this issue.”* (P7, C116)
Be Consistent	• *“The most difficult thing is consistency. Sometimes the rules change during the pandemic, and we’re following the rules. As a result, many didn’t believe it. There were also many hoaxes and misinformation. We, who are in the hospital, are also affected.”* (P7, C135)
Be Responsible	• *“As head nurses, we should be responsible for anything in the unit.”* (P7, C121) • *“… be ready at any time the staff needs and willing to sacrifice.”* (P2, C17)

## 4 Discussion

The study findings provided additional insights into the core competencies of FLNMs. The authors are aware that the main themes may not be novel, but the new dimensions may help refine the lists of FLNMs’ core competencies for the new normal.

### 4.1 Managerial core competencies

Out of the seven subthemes of the managerial core competencies, we highlight the dimensions that might be different from the existing research: 1) FLNMs should be able to establish their own reward system within their unit using their allocated budget as hospital incentives for nurses are often delayed, missed, or insufficiently implemented. However, incentives are essential for recognizing nurses’ efforts and supporting their wellness [5]; 2) FLNMs should be prepared to provide training for their staff (training of trainers), particularly during emergencies when finding external trainers becomes challenging [8]; 3) The study highlights the importance of FLNMs seeking performance assessment from middle and top nurse managers. Often, the focus is predominantly on evaluating staff nurses, making this aspect overlooked; 4) FLNMs must prioritize self-care and embrace a healthy lifestyle, a factor often disregarded but emphasized in prior research [22]; 5) FLNMs play a central role in care coordination and collaboration to improve patient outcomes, particularly in situations involving overlapping responsibilities between nurses and other professions [23]; 6) FLNMs require the scientific ability to assess and apply the evidence-based practice, staying update to address misleading information accessed by patients and families online [24]; 7) FLNMs should serve as role models, employing adaptable leadership styles and political skills. This competency is considered universal as they must lead, empower, and solve conflict [24]. However, leadership education is necessary for FLNMs [25]; 8) FLNMS are responsible for maintaining records, conducting inventory, and communicating supplies and equipment. Although this is traditionally an equipment manager’s role but identified in our study; 9) FLNMS are encouraged to be more involved in the hospital budget process, even though they lack direct decision-making authority regarding financial matters in their units consistent with prior research [26]. However, finance is considered one of the most-cited core competencies required by nurse managers [12].

### 4.2 Clinical core competencies

This study highlights the significant emphasis placed on clinical core competencies as managerial core competencies among FLNMs. The essential competencies include emergency skills, critical care, case management, infection control, and prevention. The rationale behind incorporating these core competencies is that FLNMs often provide direct patient care during nursing staff shortages. Furthermore, these core competencies are crucial for training and coaching nurses within the unit.

Hospital disaster or crisis management is an imperative skill set for FLNMs to enhance preparedness, as corroborated by prior research studies [5,9]. Additionally, lessons from the pandemic emphasize the need for a comprehensive preparedness plan to respond to future disasters effectively. However, successful disaster management requires a multidisciplinary approach [27] and emphasizes the importance of interpersonal communication competence among FLNMs [8].

Apart from basic clinical patient care, FLNMs need a strong grasp of medication management, including drug drips, dosage calculation, and handling potential errors, as nurses serve as the final defense in medication administration [28]. With higher patient acuity, nurses administer approximately ten medication doses daily, emphasizing the need for FLNMs to equip nurses to handle complex medication tasks [29].

### 4.3 Technological core competencies

The technological core competencies can be seen as a mediated factor in delivering the roles and functions in management and clinical practice. The participants emphasized using WhatsApp for communication and Zoom meetings for virtual collaboration. These findings highlight the importance of FLNMs’ understanding and proficiency in utilizing these tools to support their work. This aligns with a previous study [30] that stated that technologies, such as instant messaging, document sharing, video conferencing, social media, and other technologies, mediated performance and social influence in healthcare settings. Additionally, integrating hospital data through electronic health records interoperability is crucial for enhancing care quality [31].

### 4.4 Personality traits

Personality traits are a subset of socio-emotional skills that seem to explain why people in leadership positions act the way they do [32]. This study identified six personality traits for FLNMs: bravery, speed, patience, optimism, consistency, and responsibility. First, FLNMs must be brave in critical situations, prioritizing principles and fostering a safer environment. They inspire and enable others through professional behaviors and values [33]. Second, FLNMs must possess speed, respond quickly, be proactive, and execute tasks efficiently. Referred to as “speed leadership,” this emphasizes completing tasks within a limited timeframe and delivering optimal results [34]. Third, patience is essential, particularly in the face of challenges and societal issues related to the pandemic. Fourth, optimism is vital, enabling FLNMs to navigate challenges, find opportunities, and persevere in nursing practice. While not everyone may naturally possess optimism, it can be nurtured and developed [35]. Fifth, consistency plays a critical role in FLNMs’ work, providing clear and consistent messages to foster trust and stability among staff [36]. Finally, responsibility emerges as a vital trait for FLNMs for managing and evaluating staff performance, teaching, coaching, empowering their team [37], and being present and creating a support network [5].

### 4.5 Implications of the study

First, the identified core competencies can serve as a valuable guideline for updating FLNMs’ core competencies, allowing organizations to align their management training programs accordingly. Second, the findings can contribute to developing an objective tool for assessing FLNMs’ core competencies. Third, the identified core competencies highlight areas for hospitals to provide training programs, including leadership, disaster/crisis management, medication management, technology, and professional/personal development, to equip FLNMs with the necessary tools for success. Fourth, implementing performance assessments designed for FLNMs is recommended to ensure and maintain quality in their roles. Fifth, hospital managers are encouraged to involve FLNMs in financial management for their respective units, enabling them to contribute expertise, promote ownership and accountability, and make informed resource allocation and budgeting decisions.

### 4.6 Limitations of the study and recommendations for future research

The study findings were limited to a single hospital setting, which may not represent all FLNMs in Indonesia. Further research is needed to validate the core competencies across multiple hospitals or healthcare settings. Future studies should evaluate the core competencies’ construct validity and psychometric properties to enhance their reliability using a larger sample of FLNMs. Comparative studies between FLNMs in different regions or countries can also provide insights into potential cultural or contextual variations in core competencies.

## 5 Conclusion

As the world transitions to an endemic phase of COVID-19, the recovery process is expected to be prolonged, spanning several years. New challenges will arise throughout this period, prompting the development and implementation of innovative solutions. While this study may not discover a flawless approach to identifying all fundamental skills, it does propose a novel model of core competencies, providing a starting point for FLNMs. FLNMs have self-development goals, and this new competency model offers a standard for hospital managers to select competent nurse managers. Ultimately, this has a positive impact on healthcare organizations in the new normal era. The core competencies identified in this study encompass managerial, clinical, and technological skills and personal traits, all of which are crucial for FLNMs in this evolving landscape. It is important to note that further research is necessary to validate and confirm these core competencies in future studies.

## Funding

This study was supported by Second Century Fund (C2F), 10.13039/501100002873Chulalongkorn University, Bangkok, Thailand. Funder has no role of study design, data analysis, interpretation, and drafting the manuscript.

## Data availability statement

The data generated during and/or analyzed during the current study are available from the corresponding author upon reasonable request.

## CRediT authorship contribution statement

**Joko Gunawan:** Conceptualization, Methodology, Validation, Formal analysis, Investigation, Data curation, Writing – original draft, Writing – review & editing, Project administration, Funding acquisition. **Yupin Aungsuroch:** Conceptualization, Methodology, Validation, Formal analysis, Investigation, Data curation, Writing – original draft, Writing – review & editing, Project administration, Supervision, Funding acquisition. **Mary L. Fisher:** Conceptualization, Methodology, Validation, Formal analysis, Data curation, Writing – review & editing, Supervision. **Colleen Marzilli:** Conceptualization, Methodology, Validation, Formal analysis, Data curation, Writing – review & editing. **Nazliansyah:** Methodology, Validation, Formal analysis, Investigation, Writing – review & editing. **Ety Hastuti:** Methodology, Validation, Formal analysis, Investigation, Writing – review & editing.

## Declaration of competing interest

The authors declare that they have no known competing financial interests or personal relationships that could have appeared to influence the work reported in this paper.
