# Supplementary material for: Refining core competencies of first-line nurse managers in the hospital context: A qualitative study
Source: Int J Nurs Sci. 2023 Aug 10;10(4):492–502. doi: 10.1016/j.ijnss.2023.08.001 (PMC10667124; doi:10.1016/j.ijnss.2023.08.001)
Supplement: Multimedia component 1 [file mmc1.docx]

加强医院一线护士长的核心能力：质性研究

Joko Gunawan, Yupin Aungsuroch, Mary L. Fisher, Colleen Marzilli, Nazliansyah, Ety Hastuti

【摘要】

目的 在新常态背景下要应对不断出现的新挑战，临床一线护士长需要提高技能，以有效履行职责、发挥多重作用，亟需完善新形势下其核心能力指南或标准。本研究旨在探讨后疫情时代背景下印度尼西亚医院一线护理管理者的核心能力。

方法 采用质性描述性研究。2022年1—8月，在印度尼西亚的1所公立医院选择7名有新型冠状病毒疫情期间护理管理经验的一线护士长进行面对面访谈。对访谈内容进行录音，逐字逐句转录，并通过重新聆听进行验证。采用主题分析法对访谈资料进行分析。

结果 加强医院一线护理管理者的核心能力包括4个方面：管理核心能力，临床核心能力，科技核心能力，以及社会情感技能/个人特质（包括勇敢、迅捷、耐心、乐观、坚持和责任心）。

结论 研究结果表明，一线护士长的管理和临床核心能力必须保持一致，科技核心能力是两者的中间组成部分。个人特质对于一线护士长至关重要，因为这是他们的其他3个核心能力和成功的基础。

【关键词】临床能力；核心能力；护士长；印度尼西亚；社会技能

通信作者: Yupin Aungsuroch, E-mail: yaungsuroch@gmail.com
